# 

**Published:** 1920-04-10

**Authors:** 


### TUBERCULOSIS NOTES.

### Natural Heliotherapy.

The weather this spring has given the possibility of an unwontedly long "sunbathing season", for an English winter, unlike an Alpine one, makes open-air undressing an impracticability. Daylight saving, which suits these latitudes, at any rate, most excellently further prolongs the available hours of sunshine. If surgical tuberculosis cases can be given a little hut accommodation in the garden, or even yard, they may often get some benefit even from home treatment thus. The difficulty often lies, however, in the very simplicity of the means of treatment and its technique. To recline in bathing costume in the sun seems such a primitive thing, so unsophiscated, that the working-class mother is hard to convert to the view that it will really help to bring down the swelling in her child's elbow or knee. But, given a greater share to science in the national culture (and sanitary science bulks enormously in the popular eye to what it did twenty years ago), this matter will tend to right itself with time.

### Grants for Sanatoria.

It is announced in a Ministry of Health circular to sanitary authorities that, in order to secure quickly new additional tuberculosis accommodation, the Treasury have agreed to make grants at the rate of £180 per bed, subject to a maximum grant of three-fifths of the total cost. The conditions are that plans must be submitted to the Ministry before the end of next June, and that all buildings must be completed at latest within two and a half years from the date of the Ministry's approval of the work, unless (a weakening provision!) an extension of time is granted. In aid of working expenses, loan interest, etc., an annual allowance of half the net deficit on the tuberculosis maintenance account will be available.

### Gifts and Bequests.

MR. H. R. KINNEAR, formerly of Shanghai, has bequeathed £1,000 to King Edward's Hospital Fund.

MAJOR ELLIS WARD, of Bath, left £50 each to the Royal United Hospital, Bath, and the General Hospital, Tunbridge Wells.

MR. J. T SUTCLIFFE, of Hebden Bridge, Yorkshire, solicitor, has left £1,000 to the Royal Halifax Infirmary and £500 to the Hebden Bridge Nursing Institute.

AFTER the death of his wife, Mr. L. C. Wakefield has directed in his will that £300 each shall be paid to King Edward's Hospital Fund, St Mary's Hospital, Paddington, the Paddington Green Children's Hospital, and the National Benevolent Institution.

THE Belgrave Hospital for Children, Clapham Road, has received an anonymous gift of £750.

THE Chelsea Hospital for Women has received a bequest of £200 from the late Mrs. Alfred Goldsmid.

MR. JOHN ANDERSON, who left £186,004, bequeathed the residue of his property to the Royal Infirmary, the Western Infirmary, and the Victoria Infirmary, Glasgow.

ADDENBROOKE'S HOSPITAL, CAMBRIDGE, has received a legacy of £300 (free of duty) from the late Miss Elizabeth Watson, of Cambridge, and £1,000 for the endowment of a bed "In memory of the late Alan James Jackson, who fell in the war on April 22, 1915," who prior to joining the Army was a student at Caius College, Cambridge.

